# Complete Genome Sequence of Community-Acquired *Klebsiella pneumoniae* KP36, a Strain Isolated from a Patient with an Upper Urinary Tract Infection

**DOI:** 10.1128/genomeA.01403-16

**Published:** 2016-12-15

**Authors:** Wei-Hung Lin, Po-Xing Zheng, Tsunglin Liu, Chin-Chung Tseng, Wei-Chu Chen, Ming-Cheng Wang, Jiunn-Jong Wu

**Affiliations:** a Department of Internal Medicine, National Cheng Kung University Hospital, College of Medicine, National Cheng Kung University, Tainan, Taiwan; b Center of Infectious Disease and Signaling Research, National Cheng Kung University, Tainan, Taiwan; c Department of Biotechnology and Bioindustry Sciences, National Cheng Kung University, Tainan, Taiwan; d Department of Biotechnology and Laboratory Science in Medicine, School of Biomedical Science and Engineering, National Yang Ming University, Taipei, Taiwan

## Abstract

Here, we announce the complete genome sequence of *Klebsiella pneumoniae* KP36, a strain isolated from a patient with a severe community-acquired urinary tract infection. This genome provides insights into the pathogenesis of a pandemic *K. pneumoniae* strain from a community-acquired urinary tract infection.

## GENOME ANNOUNCEMENT

Urinary tract infections (UTIs) are a serious health problem affecting millions of people each year. UTIs usually start as bladder infections and often ascend to affect the kidneys and ultimately cause renal failure, bacteremia, severe sepsis, and even mortality (1, 2). *Escherichia coli* is the most common etiologic agent of community-acquired UTI (3, 4). The second most common uropathogen in UTI, *Klebsiella pneumoniae*, receives much less attention (5). *K. pneumoniae* is a Gram-negative opportunistic bacterium belonging to the family *Enterobacteriaceae* (6). Normally, *K. pneumoniae* can be found in the stools of healthy people (7). However, in the community setting, *K. pneumoniae* infection could lead to diverse diseases, including pneumonia, UTI, and purulent abscesses at various sites. Many genomes of *K. pneumoniae* have been determined; however, a genome of *K. pneumoniae* isolated from a UTI was still missing.

*K. pneumoniae* strain KP36 was isolated from a urine sample of a clinical patient with community-acquired upper UTI (also called acute pyelonephritis). Before seeking medical advice, the female patient was relatively healthy without comorbidity. She was admitted due to severe symptoms: fever, pyuria, and knocking pain. Because genome database entries of *K. pneumoniae* from community-acquired UTI were not available, KP36 was chosen here for sequence analysis.

*K. pneumoniae* strain KP36 was cultured overnight in LB broth at 37°C. The genomic DNA was extracted and sequenced on the PacBio RS II platform (Pacific Biosciences, USA). Library preparation and sequencing were performed at Genomics (Taipei, Taiwan). Briefly, a 20-kb library was constructed, and a total of 189,711 reads were obtained with a mean read length of 7,718 bp. Reads were assembled using HGAP version 3.0 (8), which returned five contigs. One short contig (23,273 bp) was discarded because the assembly was problematic. For each of the remaining four contigs, the head segment was almost identical to the tail segment, indicating a circular nature of the contigs. Therefore, the redundant segments were removed. In addition, the starting positions of the circular contigs were adjusted to match those of the *K. pneumoniae* HS11286 genome obtained from NCBI. The resulting assembly of *K. pneumoniae* strain KP36 consisted of one circular chromosome and three circular plasmids; the genome size was 5,759,206 bp and the G+C content was 57.0%. Genome annotation was done using the NCBI Prokaryotic Genome Annotation Pipeline (9), which identified 5,671 genes, 84 pseudogenes, 25 rRNAs, 87 tRNAs, and 15 noncoding RNAs. The genome will facilitate exploration of the role of *K. pneumoniae* in community-acquired UTI.

### Accession number(s).

The complete genome sequences of *K. pneumoniae* strain KP36 have been deposited to the NCBI database under the accession numbers CP017385 (chromosome), CP017386 (plasmid 1), CP017387 (plasmid 2), and CP017388 (plasmid 3).
